# Environmental Impacts of the Use of Ecosystem Services: Case Study of Birdwatching

**DOI:** 10.1007/s00267-014-0317-8

**Published:** 2014-07-04

**Authors:** Jakub Kronenberg

**Affiliations:** Department of International Economics, University of Lodz, POW 3/5, 90-255 Lodz, Poland

**Keywords:** Indirect environmental impacts, Sustainable consumption, Reasonable consumption, Rebound effect, Birdwatching, Birding

## Abstract

The main reason for promoting the concept of ecosystem services lies in its potential to contribute to environmental conservation. Highlighting the benefits derived from ecosystems fosters an understanding of humans’ dependence on nature, as users of ecosystem services. However, the act of using ecosystem services may not be environmentally neutral. As with the use of products and services generated within an economy, the use of ecosystem services may lead to unintended environmental consequences throughout the ‘ecosystem services supply chain.’ This article puts forward a framework for analyzing environmental impacts related to the use of ecosystem services, indicating five categories of impact: (1) direct impacts (directly limiting the service’s future availability); and four categories of indirect impacts, i.e., on broader ecosystem structures and processes, which can ultimately also affect the initial service: (2) impacts related to managing ecosystems to maximize the delivery of selected services (affecting ecosystems’ capacity to provide other services); (3) impacts associated with accessing ecosystems to use their services (affecting other ecosystem components); (4) additional consumption of products, infrastructure or services required to use a selected ecosystem service, and their life-cycle environmental impacts; and (5) broader impacts on the society as a whole (environmental awareness of ecosystem service users and other stakeholders). To test the usefulness of this framework, the article uses the case study of birdwatching, which demonstrates all of the above categories of impacts. The article justifies the need for a broader consideration of environmental impacts related to the use of ecosystem services.

## Introduction

The concept of ecosystem services has recently become a major conceptual framework for discussing economy–society–environment interactions. To promote conservation, the environment has been fitted into the dominant paradigms and language of economics, services and values (Daily 1997; ten Brink 2011). In this way, the concept of ecosystem services was meant to be a ‘mind changer,’ suggesting that destroying the environment runs counter to humans’ interests. To a significant extent, this has proved successful, especially with large-scale projects, which used the concept of ecosystem services for ecosystem assessments (MEA 2005; UK NEA 2011) and broader discussions on the economic significance of nature (ten Brink 2011). The concept of ecosystem services is now used in many political documents and by different political bodies (including governments and international organizations). All of this is expected to translate into a broader understanding of our dependence on nature (for anthropocentric and utilitarian reasons), eventually leading to changes in human behavior resulting in positive environmental impacts.

Ecosystem services are defined as the benefits that people derive from nature (MEA 2005). This is a powerful metaphor, comparing the benefits, which are available thanks to certain ecosystem structures and processes, or the so-called natural capital (Costanza and Daly 1992), with those produced within an economy thanks to the stock of human-made capital. If we take this metaphor further, we can refer to the process of deriving these benefits as the use (or consumption) of ecosystem services. ‘Ecosystem service users,’ i.e., individuals, groups of people or whole societies, benefit from using specific services that the environment provides (e.g., Plieninger et al. 2012). The idea of use of ecosystem services is particularly evident when a transaction is arranged within which the user pays for the delivery of a service, as described in the burgeoning literature on payments for ecosystem services (Schomers and Matzdorf 2013), or when any other arrangement is made for the user to reveal his/her willingness to pay for a given service (Czajkowski et al. 2014).

Of course, in most cases, users still do not pay for the ecosystem services that they use; in fact, the use of an ecosystem service may not require any action whatsoever on the part of the user. In other circumstances, the use of ecosystem services requires some action, either in the form of managing the environment to maximize the delivery of selected services, or simply accessing (physically entering) ecosystems in order to use a given service (which sometimes involves acquiring physical goods from ecosystems). In some cases, the use of a service requires additional products, such as harvesting equipment in the case of many provisioning services, or a viewing platform in the case of scenic beauty. There are different requirements in terms of what a user has to do to use different types of ecosystem services and these activities may overlap (Table 1). Also, in Table 1, the four categories of ecosystem services are treated as aggregates, while in reality, there may be important differences between the specific services within each category.

**Table 1:** Different categories of use of ecosystem services (++ particularly relevant; + somewhat relevant)

Ecosystem service category (MEA 2005)	What do the users typically do to use services of this category?				
	Managing ecosystems to maximize the delivery of selected services	Accessing ecosystems	Acquiring physical goods from ecosystems	Using other goods and services	No action
Provisioning (goods directly acquired from nature, e.g., food, freshwater and timber)	++	++	++	++	
Regulating (control of natural processes, e.g., air quality regulation, carbon sequestration and pollination)	++	+			++
Cultural (non-material contributions to human well-being, e.g., recreation, esthetics and inspiration)	++	++	+	++	+
Supporting (natural processes crucial for the delivery of other services, e.g., habitats, primary production and nutrient cycling)	+				++

Altogether the different examples of what users need to do to use ecosystem services reveal that ecosystem services are not produced by ecosystems only, but by social–ecological systems. This was underlined in the Millennium Ecosystem Assessment (2005), which referred to mediation of the relationship between ecosystem services and human well-being by socioeconomic factors (such as access to manufactured, human and social capital). Some authors even suggest that the ‘provision of ecosystem services is determined by human agency, not ecosystem functions’ (Spangenberg et al. 2014) and discuss the ‘social production of ecosystem services’ (Ernstson 2013). This co-production of ecosystem services involves material, institutional and social dimensions, with the material and social ones being of interest from the point of view of the framework developed in the present article.

The metaphor of ecosystem services has been widely used, but it has also raised concern, among other reasons because it conceals the complexity of economy–society–environment interactions (Norgaard 2010; Peterson et al. 2010). Indeed, metaphors allow us to look at things from a new perspective, but they ‘also provide a partial, or biased, perception of the referents. They let us see the similarities, but they neglect the differences which divide them’ (Kronenberg 2007a). In comparing ecosystem services with those provided within an economy, we do so on the basis of certain features, which are not always exactly similar. However, the more similarities we find, the more valid a given metaphor seems to be. An apparently overlooked similarity between ecosystem services and ‘business’ services is the way both types of services generate environmental impacts.

Products and services generated within an economy are associated with unavoidable—even if unintended—environmental consequences (Baumgärtner et al. 2001). Environmental impacts may emerge at different stages of those products’ and services’ life cycles, or—in other words—at the different stages within the relevant supply chains (Rosenblum et al. 2000; Tukker and Jansen 2006; Kronenberg 2007a). These stages include the use phase, during which additional—indirect—environmental impacts emerge, such as those related to the production of other goods and services, which are necessary to use the one analyzed. To continue the metaphor, if natural capital provides us with services, their generation and use are also likely to produce additional environmental impacts throughout those services’ life cycles. The scale of these impacts depends on the extent of intervention required to use a given ecosystem service, and on the users’ willingness to change their behavior to limit those impacts. These issues have already been discussed extensively within the sustainable consumption literature (c.f. Reisch and Røpke 2004; Jackson 2005), which demonstrates that we can use sustainable consumption as a context to analyze the use (or ‘consumption’) of ecosystem services. Indeed, sustainability is the ultimate objective of the discussion on ecosystem services.

This article puts forward a general framework for analyzing the environmental impacts of the use of ecosystem services and suggests that much attention should be paid to indirect effects. Indirect effects may undermine the initial conservation objectives associated with the introduction of more environmentally friendly products or the supposedly environmentally friendly forms of ecosystem use. In some cases, referring to the concept of ‘ecosystem services’ for the benefit of environmental conservation may lead to adverse consequences. This may happen especially when information on the availability of benefits to be derived from nature leads to altering the environment to facilitate the generation of the desired service, or to increased numbers of users and a resultant increase in indirect environmental effects. Clearly, information and awareness mediate all other environmental impacts.

The proposed approach is novel inasmuch as environmental impacts related to ecosystem services have only been studied from the perspective of how various production processes or economic activities might affect ecosystem services themselves. Initiatives of this kind include incorporating the impacts on ecosystem services into life-cycle assessments (LCAs) (Zhang et al. 2010; Koellner et al. 2011) and environmental impact assessments (EIAs) (OECD 2008; Landsberg et al. 2011). While the use of some ecosystem services can be seen as an economic activity, there are many other instances, which do not fall into this category, and discussions on ecosystem services are usually accompanied by only positive environmental connotations. The main contribution of this article is to highlight the complex dual (positive and negative) nature of the use of ecosystem services, in particular the importance of indirect effects of the use of ecosystem services, which has not been considered in impact assessments so far. For example, although Landsberg et al. (2011) focused on how the assessed projects affected the indirect drivers of ecosystem change, such as changes in local population numbers or incomes and the related demand for ecosystem services, the demand for ecosystem services itself was not translated into different categories of environmental impacts. This is where the present article steps in.

To explore these issues, we examine a case study of ecosystem services related to birds. Birds are perceived as agents of ecosystem services (Whelan et al. 2008; Wenny et al. 2011), and their existence depends on ecosystem structures and processes. Although birds contribute to a broad array of human needs, the focus here is on cultural services related to birdwatching. Similar to other forms of ecosystem-based recreation, birdwatching provides an obvious example of a service that could not be used without the additional indirect (associated) consumption of travel, birdwatching equipment etc., and it requires the birdwatcher to be present in an ecosystem. Because of its increasing popularity, birdwatching provides a good example of a potential rebound effect (e.g., a new environmentally friendly solution is used to such an extent that the environmental impact related to all instances of its use is higher than the aggregated environmental impact of the previously used solution). Indeed, birdwatching or at least some of its forms may turn out not to be sustainable in the broader sense—taking into consideration its general impacts, not just on birds but on the environment on which birds depend.

To help analyze such complex issues, a framework is put forward in the following section, along with some information on the materials used for this study. Then, the framework is illustrated with a case study of birdwatching, linking the modern context of birdwatching with the direct and indirect aspects of ecosystem services use. The final section concludes and discusses the broader applicability of the proposed framework and the implications from the case study of birdwatching.

## Methods and Materials

To link the use of ecosystem services with the classical definition of sustainable development, we need to consider whether current ecosystem service users do not preclude the ability of future ecosystem service users to satisfy their needs. Indeed, in some circumstances, the use of ecosystem services limits the capacity of ecosystems to provide these services in the future. This issue is very similar to the traditional definition of sustainable consumption as ‘the search for consumption patterns that reduce human pressure on the environment’ (van den Bergh and Ferrer-i-Carbonell 1999). The ability of future generations to use ecosystem services depends on the state of the environment; thus, we need to understand whether our use of this environment does or does not undermine its future use. The concept of ecosystem services makes this particularly clear, and it facilitates further analysis because the different forms of using the environment are distinguished as separate services, the environmental effects of the use of which can be relatively easily traced.

**Fig. 1:**
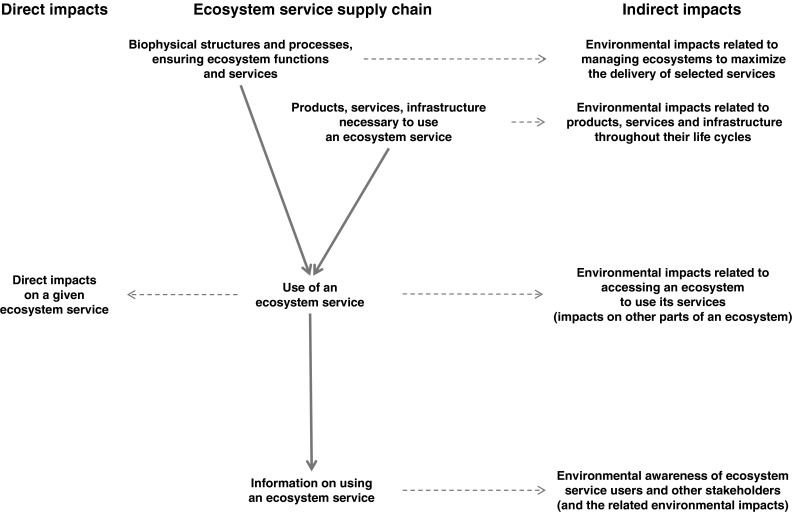
Framework to assess environmental impacts of the use of ecosystem services

The use of ecosystem services entails direct and indirect environmental impacts, which can be associated not only with the actual use of an ecosystem service, but also with other activities that this use entails (Fig. 1). The underlying metaphor suggests that altogether these activities can be called an ‘ecosystem service supply chain,’ including everything that is necessary for a service to be generated and used, its actual use, and the subsequent effects on other potential uses, through relevant information flows. Ultimately, the use of ecosystem services is driven by human behavior, either at the individual or group level (communities, companies, etc.). The framework presented below is meant to be applicable to all of such cases, and its main objective is to highlight the broader context of the use of ecosystem services and its environmental implications.

Direct impacts related to the use of an ecosystem service emerge when this use limits the service’s future availability. This is related to a potential overuse of a given service and to how this ecosystem is managed. This is most easily shown in the case of provisioning services, e.g., one cannot extract too much rubber from a tree and be able to use this tree as a source of rubber in the future. Nevertheless, overuse has also occurred in the case of other types of services, including regulating and cultural (Cairns 1997; MEA 2005). This is evident in the case of birdwatching, discussed in detail in the following section. Whereas birds are not physically consumed, birdwatchers may, by their presence, affect their numbers by lowering the attractiveness of bird sites for the birds. This demonstrates that although the act of using a given service (especially in the case of cultural ecosystem services) may not necessarily involve any form of physical consumption, it may still translate into reduced availability of this particular service in the future. Direct impacts on ecosystem services have been emphasized in different frameworks, incorporating impacts on ecosystem services into LCAs and EIAs (Zhang et al. 2010; Landsberg et al. 2011).

Nevertheless, in the case of many ecosystem services, the direct impacts of their use on their future availability are minimal. Indeed, this is reflected in the term ‘non-consumptive’ often used to denote an environmentally preferable type of use, which—unlike ‘consumptive’ use—is not making a given good or service unavailable for further uses (Tremblay 2001), as is the case with many cultural, regulating and supporting services. However, this by no means signifies that such an activity has no environmental impacts (Lemelin 2006). In most such cases, these impacts are likely to be indirect, and they refer to the different categories of use of ecosystem services, as explained in Table 2 and the following paragraphs. Indirect impacts affect other components of ecosystems (perhaps even other ecosystems) and they may eventually—indirectly—reduce an ecosystem’s capacity to provide the initial service.

**Table 2:** Importance of different categories of impacts of the use of different ecosystem services (++ particularly relevant; + somewhat relevant)

Categories of ecosystem services	Direct impacts on a given ecosystem service	Indirect impacts related to…			
		… managing ecosystems to maximize the delivery of selected services	… accessing ecosystems (on other components of an ecosystem)	… consumption of additional artifacts necessary to use a selected service	… environmental awareness of ecosystem service users and other stakeholders
Provisioning	++	++	++	++	++
Regulating		++	+		+
Cultural	++	++	++	++	++
Supporting		+			+

An ecosystem’s capacity to deliver services is limited; hence, ecosystems may be managed in order to maximize the delivery of certain services (e.g., agriculture, timber production and carbon sequestration). These management decisions produce consequences for ecosystem structures and processes. Sometimes they affect ecosystems’ capacity to provide other services, which has been discussed in the literature on trade-offs between ecosystem services (e.g., Rodríguez et al. 2006; Ruhl et al. 2007; Raudsepp-Hearne et al. 2010). For example, managing an ecosystem for carbon sequestration or for timber production may prevent the delivery of some other regulating or cultural services, which require high biodiversity. Nevertheless, most often ecosystems do not provide a single service at a time, but multiple complementary services, often called bundles of ecosystem services (Foley et al. 2005; Bennett et al. 2009). Thus, an ecosystem may be managed to deliver several services (e.g., agro-forestry programs), as opposed to a single service (e.g., plantations).

Indirect impacts are also associated with the fact that users’ presence and activities in accessing ecosystems influence other components of these ecosystems. Ultimately, albeit indirectly, they may also undermine the ecosystem’s capacity to provide the initial services, once some important biophysical structures are broken as a result of such interference. Collecting ecosystem goods (provisioning services), as well as tourism and other recreational activities, provide good examples of circumstances when the users’ presence can affect an ecosystem’s capacity to provide other services. On a larger scale, the users’ presence in ecosystems results in habitat fragmentation and other forms of ecosystem degradation.

Indirect impacts related to managing and accessing ecosystems are closely related. Indeed, in some circumstances (e.g., in the case of regulating and supporting services), access is minimal and restricted to managers, who introduce changes in an ecosystem to ensure the delivery of selected services on behalf of users. The managers’ (or their contractors’) personal impacts related to accessing ecosystems are minimal, especially compared with the consequences of the relevant management decisions (such as personal traces left by contractors managing a peat bog used for flood control, as opposed to the management decisions regarding the particular designation of a peat bog). Meanwhile, using provisioning and cultural services requires the users to access the ecosystems; hence, they are more likely to leave significant personal traces. In any case, the general mechanism is similar as it refers to introducing changes in ecosystems, affecting those ecosystems’ capacity to provide other services. In consequence, these issues can be discussed together (as in the case study of birdwatching in the following section).

Often, the use of an ecosystem service requires additional consumption of some artifacts (products, infrastructure or services). As already indicated in the introduction, using services generated within an economy entails environmental consequences. For example, whenever we visit a hairdresser or use educational services, we need resources, either to reach the service provider (transportation) or material products (from scissors to electric hair clippers, from blackboards to interactive whiteboards). All of these generate additional environmental impacts throughout their life cycles. Similar indirect environmental impacts can be generated whenever we attempt to reach and use many ecosystem services. All of these products and materials have their own life-cycle environmental impacts, which should be taken into account when assessing the use of an ecosystem service. This is especially the case of provisioning and cultural services, such as in the above examples of rubber extraction and recreation. Indeed, as Farley (2010) already indicated in this journal, ‘As all market goods must be produced from the structural elements of natural capital, and depletion of structure diminishes function, production of market goods in general must reduce the ability of the ecosystem to generate ecosystem services,’ including—indirectly—the initial service involved.

Finally, indirect effects of the use of ecosystem services may also emerge as a result of the spread of information on the availability of those ecosystem services, and on the opportunities to use them. Thus, one needs to consider the broader impacts on society as a whole related to the use of ecosystem services. The concept of ecosystem services is being ‘mainstreamed’ (Cowling et al. 2008) in order to promote better understanding and motivation for environmental conservation among the broader public. However, especially in the case of some provisioning and cultural services, such as collecting non-timber forest products or tourism, information may inspire potential new users to seek such benefits from nature. Meanwhile, too many users and too much infrastructure resulting from the popularity of a site may reduce its attractiveness and ultimately lead to its degradation. Nevertheless, information about a site which is important from the point of view of ecosystem services may also contribute to this site’s protection, including when this information is spread by the services’ users. In short, indirect impacts related to environmental awareness of ecosystem service users and other stakeholders influence all other categories of impacts.

All of the above categories of impacts need to be investigated to provide a comprehensive picture of the environmental impacts of the use of ecosystem services. Proper assessment of the first three categories requires an understanding of the ecological context of the ecosystem service in question. The fourth category is linked to what we already know about environmental impacts of products, drawing from ecological economics and industrial ecology. The final category is linked to the motivations for sustainable or environmentally friendly behavior, in particular those that show that the environment can be protected for one’s own interests.

The applicability of the above general framework to analyze the environmental impacts of ecosystem service use will be tested in the following section, using the example of birdwatching. Like all other examples of tourism, birdwatching provides a good illustration of direct and indirect environmental effects (Gössling 2002). Few researchers have actually looked at the potential benefits and problems related to birdwatching on a broader scale (Sekercioglu 2002). However, much insight can be gained into these issues through the literature on the social side of birdwatching, which has recently proliferated, with not only academic articles but also popular books published on the behavior and habits of birdwatchers (e.g., Cocker 2002; Koeppel 2006; Weidensaul 2007). Such accounts were also available earlier (e.g., Hickling 1983; Gibbons and Strom 1988), but the recent decade has witnessed a surge of such publications. This has been accompanied by a further number of books devoted to the cultural meaning of birds, within which birdwatching plays an important part (e.g., Cocker and Mabey 2005; Collar et al. 2007; Mynott 2009). Indeed, the availability of such publications illustrates the growing popularity of birds and birdwatching, and at the same time the growth of birdwatching-related consumption. The review of this literature provided useful material for this study, complemented with the author’s participant observation.

## Environmental Impacts of Birdwatching

Birdwatching is a relatively new form of activity for large sectors of society, emerging since the beginning of the twentieth century. Before that, it was reserved for specialists (Bircham 2007). From the very beginning of this activity, birdwatchers condemned killing birds for various consumptive uses, promoting instead a new, *non*-*consumptive* approach of just watching birds. Today, birdwatching encompasses broad spheres of societies in several developed countries, particularly in the USA and the UK. In the USA, 46.7 million people observed birds in 2011, with 88 % of them observing wild birds around home, and 38 % on trips away from home (U.S. FWS 2012). In the UK, it has been estimated that more than 6 million people engage in birdwatching every couple of weeks (Kellaway 2009; CBI 2011). Furthermore, with more people traveling to remote locations, there are increasing opportunities for local guides to show them exotic birds, and even if these tourists are not formally classified as birdwatchers, they become involved in this activity.

On the one hand, this broad interest translates into support for bird conservation and reintroduction programs. On the other hand, it brings about increased penetration of ecosystems and increased consumption of goods and services that are perceived as birdwatchers’ indispensable attributes, such as optical equipment, books, travel, participation in dedicated workshops and festivals and birdwatching holidays. Thus, two perspectives on birdwatching can be contrasted: the idealistic feeling of an intimate bond with nature and mainstream consumption, where birdwatching is just another sector of the economy. Birdwatching tends to be presented as ‘one of the most ecologically sound and sustainable of versions of wildlife tourism’ (Connell 2009), and at the same time ‘one of the fastest growing wildlife-based activities’ (Roe et al. 1997).

An important caveat is that there are different categories of birdwatchers, and their relations to birds and the ‘use’ or ‘consumption’ of birds vary. Birdwatching is an umbrella term, encompassing all other categories. Birding tends to be more specialized and professional (Sheard 1999), although in North America, the term ‘birding’ often replaces ‘birdwatching.’ ‘Twitching’ refers to the quest to see rare species, especially those that are difficult to find, or outside of their typical geographic range (‘the obsessive art of chasing rare birds that one has not seen before, yea, unto death’ (Gooddie 2010)). This is related to ‘listing,’ whereby one maintains a list of species seen in a given area, and ‘big listing’ if one tries to see all species of birds. Of course, there are also other classifications of birdwatchers, some of which are based on how many birdwatching trips they undertake per year or on their identification skills—from casual or novice to advanced or experienced (e.g., McFarlane 1994; Hvenegaard 2002; Scott and Thigpen 2003; Scott et al. 2005). In general, the more professional and ‘obsessed’ a birdwatcher is, the more significant his/her environmental impacts are likely to be. For the sake of illustration, much of the following focuses on the environmental impacts associated with the more ‘obsessed’ group.

The key direct and indirect environmental impacts of birdwatching, analyzed according to the framework put forth in the previous section, are summarized in Table 3 and discussed in more detail in the following subsections.

**Table 3:** Direct and indirect impacts of birdwatching

Category of impact	Summary
Direct impacts	Although birdwatching does not involve physical consumption of birds, the ‘symbolic’ appropriation associated with it may still translate into direct impacts on birds (increasing pressure on birds)
Indirect impacts related to managing and accessing ecosystems	Accessing ecosystems to observe birds, birdwatchers impact not only birds and their habitats but also other components of those ecosystems. Managing land to favor birdwatching incorporates further trade-offs
Indirect impacts related to consumption of additional products and services	Modern birdwatching requires a multitude of products and services, all of which translate into environmental impacts generated by those additional products and services throughout their life cycles
Indirect impacts related to information and environmental awareness	Like the concept of ecosystem services, birdwatching is expected to enhance the understanding of human linkages with nature and to raise political support for conservation. The expected positive spillover effects refer to birdwatchers’ role in gathering scientific data, their ability to fund conservation, and increasing social support for the protection of birds. Potential negative effects include increased visitation related to information provided by those who have already accessed some sites

### Direct Impacts: Bird Consumption Redefined

Birdwatchers have always been to a large extent motivated by the esthetic appeal of birds and, like many other naturalists, they derive pleasure from their interaction with birds and more generally with nature (even if sometimes they can also feel resentment, anger and apathy). This reflects direct consumption of cultural ecosystem services: the presence of birds and opportunity to observe them. Birdwatching is seen here as a form of reconnecting to the natural world and partly serves to escape the modern, consumption-oriented society that has gone so far in detaching itself from the environment (e.g., Watson 2010). ‘To a birdwatcher, one glimpse, one moment is happiness enough,’ suggested Kellaway (2009), reflecting the emotional, spiritual, physical and mental stimulation derived from watching birds or being able to enjoy their presence (Conradie et al. 2013).

However, viewing wildlife is a visual experience and often ‘requires’ a proof. This is connected with the idea of seeking ‘souvenirs’ or ‘trophies’ which would attest that the visitor had indeed visited a given place or seen a given species. This discussion dates back at least to when Leopold (1949) observed the beginning of the commodification of nature, noting that wildlife watching rested ‘upon the idea of trophy,’ be it a landscape photograph or a close-up picture of a bird. The idea that such trophies may be more important than the experience itself has given rise to the concept of ocular consumption (Lemelin 2006).

The obsessive character of some forms of birdwatching is related to the birdwatchers’ feeling of constant non-satiation; once they see one new species, they immediately set their sights on observing another new one (Oddie 1995; Liep 2001; Koeppel 2006; Connell 2009). This resembles typical consumption behavior: ‘the dream will be carried forward and attached to some new object of desire such that the illusory pleasures may, once more, be re-experienced’ (Campbell 1987). Many birdwatchers add to this a high degree of competitiveness (Oddie 1995; Schaffner 2009). Just like all other collectors, the fewer specimens they lack to complete their ‘collection,’ the more they are likely to pay to attain the final satisfaction (Mynott 2009), be it in terms of money or effort, or even to the extent of ignoring some of the rules that they would have otherwise obeyed, e.g., with regard to protecting a bird and its habitat.

The above obsessive symbolic appropriation of birds (Liep 2001) often translates into direct impacts on bird populations. By their presence and obstinacy, birdwatchers affect the attractiveness of the breeding, migration or roosting sites for birds, flush birds, and otherwise increase the pressure on birds and their habitats (e.g., luring birds out of their hideouts and stressing them by playing their calls or exposing birds and their nests to predators). These intrusions often result in direct impacts on their mortality, productivity and behavior, as well as habitat degradation (Klein et al. 1995; Burger and Gochfeld 2001; Gill 2007; Karp and Root 2009). They are not only caused by the most determined twitchers and listers, but also by casual birdwatchers and other people visiting bird sites (Steven et al. 2011; Huhta and Sulkava 2014). Especially in less developed countries, guides tend to be particularly eager to show the birds to tourists from as close-up as possible. Because of their lack of experience or interest in bird conservation, these tourists do not object to being involved in activities that might harm nature. For these reasons it has already been suggested that people who enter bird habitats should be educated on the impacts that they cause to birds (Corre et al. 2013).

The above impacts emerge out of the visitation of areas where birds reside and constitute a symbolic consumption of birds. However, because most birdwatchers reveal concern over conservation issues related to birdwatching (Kellert 1985; Green and Jones 2010), and as various birdwatchers’ codes of ethics are promoted by the relevant organizations and bird identification guides, the potential threats that birdwatchers might bring to bird populations tend to be downplayed. Even though the publicity surrounding rare species and increased visitation sometimes lead to abandonment of a site by birds (Kemp 2000; Moss 2005), restricting public access (protecting birds from birdwatchers) is considered as the last resort and avoided because public access has very important educational and conservation potential (Gill 2007), as we shall see below.

### Indirect Impacts

#### Impacts on Other Parts of an Ecosystem (Management and Access)

Although birdwatchers use much of the same infrastructure and amenities as other tourists (roads, lodges etc.), they add to the general demand and in the case of some localities they constitute the major drivers of this demand (Connell 2009). Birdwatchers tend to go where the birds are, which means they often go where no infrastructure is available, intruding into new areas and potentially pioneering new ‘tourist trails.’ Species’ rarity has a very high influence on distance travelled and numbers of visitors (Booth et al. 2011). Thus, some authors (see, in particular, Courchamp et al. 2006) argue that ‘human perception of rarity can precipitate species extinction’ because of increased pressure on a given species and its habitat, as already indicated with respect to direct impacts on birds. Affecting habitats is relatively broadly discussed in the case of both birdwatching and ecotourism in general, with regard to the so-called environmental contradictions (e.g., Cater 1995; Isaacs 2000).

Some birdwatching trips have destinations that are considered toxic for humans. These maintain high bird populations because people have avoided the sites and birds have found them relatively tranquil. As argued by Schaffner (2009), the birdwatchers’ enthusiasm for visiting such places provides another example of their neglect of the broader context of sustainability. The above problems indicate a contradiction between some forms of ‘compulsive, acquisitive’ birdwatching and ‘the environmentalist legacies connected to the formation of birdwatching as a hobby’ (Prior and Schaffner 2011). This is partly related to the increasing numbers (and consequently impacts) of birdwatchers and to the obsessive nature of some forms of birdwatching. Indeed, even in birdwatchers’ communities, twitching is sometimes perceived as ‘anti-environmental’ (Cocker 2002).

We also need to refer to the management of birdwatching sites in a way that makes them attractive not only to birds, but also to birdwatchers. Indeed, while managing land for the benefit of birds is complementary to many ecosystem services, it competes with others (Whelan et al. 2010; Bradbury et al. 2010), and there may be trade-offs between different ecosystem management scenarios favoring different bird species (Elphick 2004). Managing land with the specific service of birdwatching in mind involves further trade-offs, and potentially also considerations of environmental justice if birdwatchers are allowed to access an area inaccessible to other stakeholders, including local populations. Indeed, as we shall see in the following subsection, because of the related economic gains, the expansion of birdwatching trails has become part of official development aid programs and aid projects carried out by environmental organizations in developing countries.

#### Impacts Related to Products, Services and Infrastructure (Induced Consumption)

Birdwatching’s evolution into a popular pastime in the beginning of the twentieth century was aided by the availability of low-cost optical equipment and bird identification books, i.e., by the development of certain markets. It developed further with a special type of bird identification guides (starting from Peterson (1934)) and with the growing popularity and availability of transportation. Big listing became possible because of the development of the international travel market. This was also related to the increase in leisure time and surplus income (Cocker 2002). Although the focus of birdwatching is on non-market goods and services (birds), a significant market has turned out to be necessary to ‘consume’ the former, and birdwatchers have become part of the commodity culture of today (Prior and Schaffner 2011). As a result, the growing popularity of birdwatching adds to environmental problems that threaten birds and bird habitats through increased birdwatching-induced consumption.

Travel generates probably the most evident environmental impact. There are six companies offering over 150 bird tours annually, and there are hundreds of smaller companies, including regional, national, local, species-specialized tour operators, lodges and many others. Extreme birdwatching (twitching, big listing) involves extreme travel, including hiring private airplanes to get to a given remote location as quickly as possible (c.f. Cocker 2002; Koeppel 2006; Mynott 2009). However, even in the case of regular and casual birdwatching, travel is important enough to indicate the economic value of birdwatching sites based on how much people spend to reach those places (c.f. Czajkowski et al. 2014).

The value of birdwatching sites or the sites of birdwatching-related events is sometimes discussed from the perspective of how much birdwatchers spend on accommodation and other local goods and services (e.g., Kerlinger and Brett 1995; Kim et al. 1998). A similar approach has been adopted by bird protection organizations in an attempt to generate support for nature conservation (RSPB 2010). Also, it was used to promote environmental protection in developing countries, indicating the potential economic gains related to preparing and maintaining a viable offer for birdwatchers (Biggs et al. 2011). Consequently, because of the predominant economic focus, the public discourse on birdwatchers concentrated on their expenses related to traveling, purchasing or consuming, resulting in ‘the vision of economic windfall for communities’ (Hill et al. 2010).

Apart from travel, accommodation and other local spending, the birdwatching-related market includes not only the traditionally important optical equipment (binoculars, telescopes, cameras) and books, but also an increasing number of other categories of goods and services (devices that enable recording of bird sounds, CDs/DVDs, dedicated television programs, exhibitions, bird feeders and birdseed, crafts, arts, mugs, toys and a multitude of other gadgets). The turnover of the birdwatching-related market has become yet another indicator of the economic value of birds. In 2006, birdwatchers in the USA spent $36 billion solely on travel and equipment, generating $82 billion in total industry output across the country (Carver 2009). Indeed, to some extent, consumption acquired a status symbol among birdwatchers—confirmed by the trips one has undertaken or equipment one possesses, leading to ‘ludicrous follies of conspicuous consumption’ (Cocker 2002). Even residential birdwatchers, who do not travel so much in search of birds but content themselves with local observations, also procure optical equipment, books, videos, birdfood and many other birdwatching-related goods and services.

Additionally, countless specialized publications, including local, regional and international magazines, offer advertising space for both avitourism and equipment. There are numerous birding festivals [more than 200 in North America alone in 2006 (Lawton 2009)], providing a platform for many commercial activities related to birdwatching. Interestingly, the commercial side of birdwatching is successfully exploited by various conservation organizations. Apart from membership fees, they brand numerous products with their names (e.g., bird guides, optical equipment and gadgets) and sell bird-related products through their own sales channels. This helps to raise funds for environmental protection while at the same time providing an opportunity to boost sales. Similarly, birdwatching-related events, such as festivals and listing competitions, often offer opportunities to promote the cause of bird/environment protection. They also help to broaden the fundraising outreach thanks to the broad media attention that they attract.

#### Impacts Related to Environmental Awareness of Ecosystem Service Users

Birdwatchers contribute to conservation, helping to build and disseminate environmental knowledge by participating in citizen science (Greenwood 2007). Because of their exposure to scientific discourse, birdwatchers tend to be better aware of environmental problems related to habitat conservation, when compared with average consumers. As a result, they are more likely to support conservation, including with membership fees in birdwatching organizations and individual donations, and motivate other people to do the same.

Being associated with economic benefits, at least to some extent, they also contribute to increasing social awareness of and support for environmental protection. In some cases, birdwatchers may have an opportunity to buy bird-related ecosystem services by paying those who protect bird habitats through various kinds of payments for ecosystem services. Birdwatching ecotourism, within which participants contribute to the preservation of ecosystems in places that they visit, serves as an example (Puhakka et al. 2011). Besides, to be able to ‘use’ birds in their own countries, birdwatchers need to consider the situation overseas, where birds migrate to and from. Sultanian and van Beukering (2008) proposed an international payment scheme within which Northern birdwatchers would pay for the protection of bird sites in the South. In a broader context, birdwatchers benefit from the fact that bird species are protected, even if they are not able to see those species themselves. This is a result of their interest in watching birds in documentaries or reading about them, or because of the potential to see those species if an opportunity arises.

From an even wider perspective, the activities of bird conservation organizations, partly facilitated by the broad outreach of birdwatching, have led broader spheres of society to consider birds in their decisions. Farmers have to make increasingly difficult choices, taking into consideration the impacts of their activity on bird populations. For example, when farmers attempt to reduce the population of birds seen as pests, they need to take into consideration the public opinion concerning such population control, which might affect the marketability of their produce (Blackwell et al. 2003). Similarly, consumers may be willing to pay a premium for agricultural products certified as bird-friendly (Foster and Mourato 2000; Rice 2010). This is related to the role that birdwatchers play, attracting others to birds and promoting interest in bird conservation.

To attract birdwatchers, some tourist resorts attempt to minimize their environmental impacts and set up private reserves (Sekercioglu 2002). However, although in 2014, four out of the top six global birdwatching companies mentioned conservation on their websites, compared with only one out of six in 2001 (Sekercioglu 2002), in most cases, the declared activities were very modest and restricted to funding offered to conservation projects. Minimizing the environmental impacts of the tours themselves was not emphasized. Similarly, while various ‘twitchathons’ or ‘birdathons’—whereby people compete to see the most species in a limited time—provide opportunities to raise funds for conservation and thus increase awareness of birds’ importance, they are excessively focused on competition and often neglect the broader context of environmental problems that threaten bird populations and the broader issues of ecosystem functioning on which birds depend (Schaffner 2009). Finally, the awareness of the popularity of some sites among birdwatchers can also lead to increased visitation and pressure, and increasing popularity of birdwatching can translate into increasing all of the other environmental impacts of birdwatching.

## Discussion and Conclusions

The framework put forward in this article for analyzing the environmental impacts of the use of ecosystem services highlights a useful extension of the ‘services’ metaphor. It indicates that ecosystem services go through life cycles similar to those observed in the case of products and services offered by economic agents. Thus, the use of ecosystem services not only influences the structures and processes that provide those particular services, but also—indirectly—other ecosystem components and other ecosystems.

The example of birdwatching serves as a good illustration, indicating the full spectrum of impacts that the use of an ecosystem service might generate. Indeed, inasmuch as habitat loss and fragmentation, overexploitation of resources and climate change count among the most important threats to both birds and biodiversity in general (Steven and Castley 2013), the activity of birdwatching contributes to the generation of these environmental problems and consequently impacts on birds, albeit mostly indirectly, through the birdwatchers’ consumption patterns. Meanwhile, most discussions so far on the negative impacts of birdwatching have focused on the direct impacts on the birds themselves, while the problem of birdwatching-induced consumption has rarely been discussed. If at all, it is usually perceived as positive, even by bird conservation organizations, as it frames birdwatching as a desirable contribution to economic development. Omitting this issue in environmental assessments of birdwatching (and by extension many other ecosystem services as well) is in opposition to the broad, life-cycle approach taken to analyze the environmental impacts of products. This links to the broader relationship between birdwatching and environmental awareness (the broad outreach of birdwatching can stimulate broader interest in environmental conservation, but also increased visitation of bird sites), which so far has also received relatively little attention. Indeed, various codes of conduct promoted by bird-related organizations focus on affecting birds and their habitats, pay little attention to the broader environmental issues and neglect problems related to birdwatching-induced consumption.

The proposed framework highlights the relevance of a sustainable consumption agenda to the discussions on ecosystem services. Watson (2010) explored the quality of the relationship between humans and the natural world that builds through birdwatching and identified an approach that is particularly relevant here. In essence, he called reflexive birdwatching ‘being (more) aware of the relationship between a birder’s kind and context of practice’ (Watson 2010). This links to the broader issue of ‘conscious’ or ‘reasonable’ consumption (e.g., Paavola 2001; Jackson 2005; Kronenberg 2007b). However, except for some conservation organizations’ communications, few examples of birdwatching accounts reflect this kind of broader environmental sensitivity (Schaffner 2009), and relatively few appeals have been made to the birdwatchers’ community to become ‘more vocal on behalf of the things they care about’ (Weidensaul 2007). Interestingly, although birdwatching originated as an inherently non-consumptive behavior, opposed to other, consumptive uses of birds, over time it evolved and ‘entered the mainstream of consumer culture’ (Cocker 2002). Many other ecosystem service uses have followed a similar path, especially as a result of making them part of the market, which has been advocated by many proponents of the concept of ecosystem services.

On many occasions, ecosystem services have been used as an opportunity to justify environmental degradation and continue ‘business as usual’ scenarios. Carbon sequestration provides a useful example, whereby instead of reducing emissions, many stakeholders seek opportunities to ‘buy themselves out’ of their responsibility by investing in carbon sequestration. The proposed examination of the direct and indirect environmental consequences of such use of ecosystem services helps us to understand the controversial character of such an approach to the use of nature. Even if the impacts of these specific emissions may be mitigated, the key issue here is that the existence of forests (or the use of forests for offsetting carbon emissions) warrants further emissions. In such cases, the concept of ecosystem services sanctions environmental degradation, to the extent that it can be mitigated with the use of instrumentally treated ecosystems and their services (Robertson 2004).

More broadly these problems, and the ability to view them in the framework of direct and indirect environmental impacts, reveal a larger problem with the concept of ecosystem services, i.e., its anthropocentric and utilitarian character. Hence, the framework proposed allows us to look at the use of ecosystem services more broadly than the approaches taken to date, and to understand that any kind of human intervention in an ecosystem entails some negative consequences.

The proposed framework complements and broadens the recent attempts to integrate impacts on ecosystem services into various EIAs (Zhang et al. 2010; Landsberg et al. 2011). The approaches to date have looked at ecosystems as part of a supply chain, delivering what is used in economic activities. But not all forms of use of ecosystem services fall into these categories; indeed, most ecosystem services are used irrespective of traditional economic undertakings. Therefore, although all of our social and economic activities ultimately depend on ecosystem services, we do not necessarily account for the way in which our use of ecosystem services undermines the opportunities of future generations to benefit from them. The Millennium Ecosystem Assessment suggested that the ecosystems’ capacity to deliver 60 % of services was threatened, mainly due to environmental degradation in general (MEA 2005). The framework proposed in this article links the actual use of ecosystem services with the threats to those services.

While the proposed framework highlights these impacts, further research is required into how to make it operational. To some extent, it can be combined with the abovementioned assessment methods to incorporate impacts of different products, services and projects on ecosystem services, and the discussions on trade-offs between different ecosystem services (Raudsepp-Hearne et al. 2010). This would help make the proposed framework more specific in terms of identifying specific impacts on other ecosystem services. However, we need to keep in mind that so far many ecosystem services have been left out from these analyses (especially cultural ecosystem services) (Zhang et al. 2010).

The approaches put forward so far have focused only on what could be quantified. Meanwhile, on the most general level, the proposed framework does not require quantification as it serves to provide a general understanding of how our current use of ecosystem services influences our ability to use those services in the future. Of course this framework allows for quantification of these impacts, but to the maximum extent possible, a comprehensive picture of ecosystem services and their qualitative dimensions should be maintained (to escape the common problem of focusing only on what is easily quantifiable) (Karjalainen et al. 2013; Kronenberg 2014). Ultimately, the main advantage of this framework is to demonstrate the broader context of use of ecosystem services. Its limitation in this regard is that some impacts that it highlights are difficult to bring together (e.g., human awareness of environmental problems vs. managing ecosystems). In this sense, it can only serve as a general indication of impacts.

The framework could be further extended to cover environmental justice issues, which become increasingly important when we study the political ecology context of some new arrangements on the use of ecosystem services (Kosoy and Corbera 2010; Sikor 2013). Indeed, these often translate into the commodification of ecosystem services and new power relations. For example, can the use of a particular ecosystem service by a particular user or group of users affect the ability of others to benefit from this ecosystem service? However, although these issues also have environmental consequences, these are mostly social issues and so have not been included in the framework yet.
